# Ginsenoside Rb1 alleviates overtraining-induced oxidative stress in mouse liver by stimulating mitophagy via Nrf2 pathway

**DOI:** 10.3389/fnut.2026.1849386

**Published:** 2026-05-13

**Authors:** Jianing Lu, Ruotian Xu, Yuexuan Gao, Luye Zong, Daming Wang, Jing Li

**Affiliations:** 1 School of Sport Health Technology, Jilin Sport University, Changchun, Jilin, China; 2 Jilin Provincial University Key Laboratory of Athletic Training, Jilin Sport University, Changchun, Jilin, China; 3 School of Winter Sport, Jilin Sport University, Changchun, Jilin, China; 4 Graduate Office, Jilin Sport University, Changchun, Jilin, China; 5 School of Human Movement Science, Jilin Sport University, Changchun, Jilin, China

**Keywords:** ginsenoside Rb1, mitophagy, Nrf2, overtraining, oxidative stress

## Abstract

**Introduction:**

Moderate exercise induces beneficial adaptive responses in the body, whereas sustained high-intensity exercise without adequate recovery leads to overtraining syndrome (OTS), a pathological condition associated with multiple organ damage, especially liver injury. Currently, safe and effective nutritional interventions for OTS-induced liver injury remain limited. This study aimed to investigate the protective effect of ginsenoside Rb1 (Gs-Rb1), the primary bioactive component of ginseng, against OTS-induced liver injury and to elucidate its underlying molecular mechanism.

**Methods:**

We established an overtraining (OT) mouse model using ICR mice, and evaluated the hepatoprotective effect of Gs-Rb1 using histopathological observation, biochemical analysis, immunofluorescence staining, Western blot, and other related techniques. The core regulatory mechanism was verified using chloroquine (CQ, an autophagy inhibitor) and ML385 (a specific Nrf2 inhibitor).

**Results:**

OT intervention induced significant oxidative stress and liver injury in mouse liver, whereas Gs-Rb1 administration effectively alleviated liver damage, improved exercise performance, and mitigated OT-induced oxidative stress and mitochondrial impairment in the liver by activating mitophagy. Mechanistically, Gs-Rb1 promoted the nuclear translocation of nuclear factor erythroid 2-related factor 2 (Nrf2), and further activated the downstream PTEN-induced kinase 1 (PINK1)/Parkin RBR E3 ubiquitin protein ligase (Parkin) pathway to drive mitophagy. Inhibitor validation experiments further confirmed that the hepatoprotective effect of Gs-Rb1 was autophagy-dependent, and Nrf2 was a key regulator of Gs-Rb1-mediated mitophagy.

**Discussion:**

This study demonstrates that Gs-Rb1 ameliorates OT-induced oxidative stress in mouse liver via Nrf2-dependent mitophagy. Our findings indicate that targeting mitophagy via the Nrf2 pathway represents a promising nutritional strategy to alleviate overtraining-related liver injury, providing a theoretical basis for the application of natural bioactive compounds such as Gs-Rb1 in the field of sports nutrition and health promotion.

## 1 Introduction

As competition in international sports intensifies, athletes often increase training intensity and frequency in pursuit of better performance. Prolonged high-intensity training with insufficient recovery can induce OTS, a condition that severely impacts both daily life and training, potentially leading to premature termination of an athlete’s career. Studies have shown that nearly 64% of elite athletes, particularly those in endurance sports such as cycling, rowing, and swimming, experience OTS at least once (1). Recent research further indicated that the risk of OTS may also rise significantly during individual or even low-intensity exercise, suggesting that overtraining is likely an underrecognized phenomenon within the scientific community (2). In recent years, with the introduction of the concept of “scientific training,” post-exercise fatigue recovery and injury prevention have become hot topics in sports science research.

As one of the most functionally complex organs in mammals, the liver plays a pivotal role in sustaining systemic homeostasis, regulating exercise performance, and maintaining blood glucose balance via hepatic gluconeogenesis. Previous work has confirmed that hepatic glucose production is a key determinant of exercise capacity by sustaining circulating glucose levels to meet the energy demands of contracting skeletal muscle (3). Therefore, when exercise-induced liver damage occurs, it often results in diminished physical performance and premature onset of exercise-induced fatigue. Accumulating studies have suggested that oxidative stress is a key pathological driver of exercise-induced liver injury, which may serve as a critical link between excessive overtraining, hepatic dysfunction, and impaired athletic performance.

Reactive oxygen and nitrogen species (RONS) are highly unstable and reactive, easily stealing electrons from other molecules to stabilize themselves, leading to oxidative stress damage in the body. In 1982, Davies et al. (4) first demonstrated through electron spin resonance spectroscopy that high-intensity exercise increases the production of free radicals. Other studies confirmed that exhaustive exercise or overtraining (where athletes engage in high-intensity exercise with insufficient recovery) leads to increased RONS production (5, 6). Liu et al. (7) reported that swimming training in rats increases free radicals in liver tissue, causes endoplasmic reticulum stress, cell membrane damage, and impairs normal liver function. These findings suggest that prolonged high-intensity exercise without adequate rest (overtraining) likely causes oxidative stress damage, and that antioxidant nutritional strategies may help in recovering from exercise-induced fatigue and injury, thereby enhancing performance.

Mitochondria are the primary site of endogenous reactive oxygen species (ROS) production and are particularly vulnerable to oxidative damage (8). During hepatic oxidative stress, damaged mitochondria further disrupt redox homeostasis and drive excessive ROS generation, forming a self-amplifying vicious cycle (9, 10). Mitophagy, a conserved cellular process that regulates mitochondrial quantity and quality via targeted clearance of impaired mitochondria, is an effective approach to break this cycle and alleviate oxidative hepatocyte injury. Natural bioactive compounds have been widely investigated for their regulatory effects on mitophagy, and the core antioxidant transcription factor Nrf2 has been well-established as a key upstream regulator of mitophagy and mitochondrial homeostasis (11). Thus, targeted modulation of the Nrf2-mitophagy axis represents a promising nutritional strategy to mitigate oxidative stress-related liver injury.

Gs-Rb1, the most abundant bioactive compound in ginseng, has been shown to exhibit antioxidant, anti-inflammatory, and lipid metabolism-regulating effects (12–16). Its potent antioxidant capacity, characterized by free radical scavenging and endogenous antioxidant enzyme enhancement, has been confirmed to mediate tissue-protective effects in multiple systems including the cardiovascular and nervous systems (17). In the liver specifically, recent studies have shown that Gs-Rb1 alleviates alcohol-induced hepatic lipid accumulation and oxidative stress, with partial restoration of glutathione levels (18). Our previous studies have demonstrated that Gs-Rb1 ameliorates toxic compound-induced lipid metabolism disorders and hepatocyte apoptosis via activating autophagy (19, 20). Building on these findings, we hypothesize that Gs-Rb1 mitigates chronic overtraining-induced hepatic oxidative stress injury and improves exercise performance via activating Nrf2-mediated mitophagy.

Most existing exercise-related studies focus on skeletal muscle adaptation, while neglecting the regulatory mechanism of hepatic metabolic homeostasis (the core hub of exercise energy metabolism) during chronic overtraining. Meanwhile, the protective effect of Gs-Rb1 against overtraining-induced liver injury and its underlying mechanism remain largely unelucidated. Therefore, this study aims to systematically investigate the protective effect of Gs-Rb1 against chronic overtraining-induced liver injury in mice, and clarify its core regulatory mechanism targeting the Nrf2-mitophagy axis.

## 2 Materials and methods

### 2.1 Reagents

Gs-Rb1 was purchased from Desite Biotechnology Co., Ltd. (Chengdu, China), while chloroquine diphosphate salt (CQ) and ML385 were obtained from Sigma-Aldrich (Missouri, USA). The purity of all reagents was over 98%. Antibodies used in the Western blotting experiments were purchased from Proteintech (Chicago, USA), and the primary antibodies for PINK1 and LC3 used in the immunofluorescence co-localization experiments were obtained from Abcam (Cambridge, UK) and Bioss (FoTan, Hong Kong), respectively. Secondary antibodies were sourced from CST (Massachusetts, USA).

### 2.2 Animal grouping and treatment

**Figure 1:**
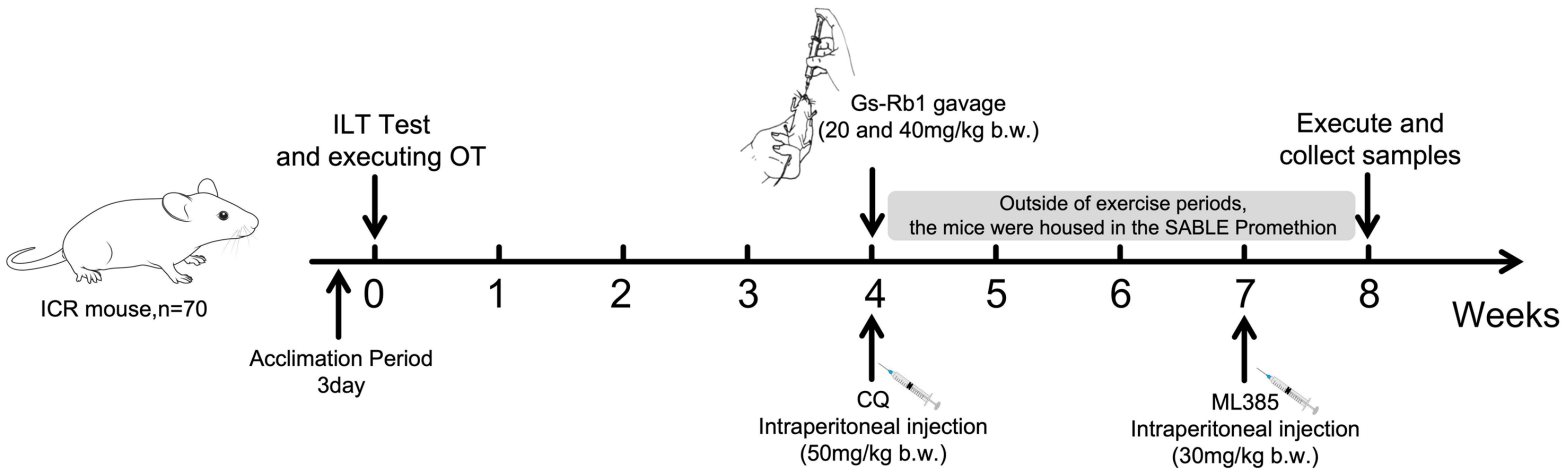
Experimental workflow.

Eight-week-old male ICR mice were obtained from Liaoning Changsheng Biotechnology Co., Ltd. (Benxi, China). All animal experimental protocols were approved by the Ethics Committee for Scientific Research at Jilin Sports University. Mice were housed in groups (5 per cage) with ad libitum access to food and water. They were fed a standard chow diet (SCD) free of antibiotics. The environment was maintained at a temperature of 24 ± 1 °C, with a relative humidity of 40–80%, and a 12-h light/dark cycle. Body weight was measured once every 7 days. After a 7-day adaptive feeding period, the mice were randomly assigned to seven groups (*n* = 10 per group) via a standardized randomization procedure: a unique random number for each mouse was generated using the RAND() function in Microsoft Excel by an independent researcher blinded to the experiment; all random numbers were sorted in ascending order, and mice were sequentially and evenly allocated to the 7 groups (10 mice per group) strictly following the sorted sequence. The experiment lasted 56 days. The groups received different exercise and nutritional interventions: Control group, Gs-Rb1 (high) (40 mg/kg b.w.) group, OT group, OT + Gs-Rb1 (low) (20 mg/kg b.w.) group, OT + Gs-Rb1 (high) (40 mg/kg b.w.) group, OT + Gs-Rb1 (high) (40 mg/kg b.w.) + CQ (50 mg/kg b.w.) group, and OT + Gs-Rb1 (high) (40 mg/kg b.w.) + ML385 (30 mg/kg b.w.) group. Gs-Rb1 was administered via gavage, while CQ and ML385 were injected intraperitoneally (The overall experimental workflow is shown in Figure 1). To alleviate animal suffering, isoflurane anesthesia was administered when necessary during the experimental procedures.

### 2.3 Incremental load test (ILT)

An incremental load test was conducted at the start of the experiment and after the completion of each week’s training to assess EV as a measure of exercise performance. The test was performed using a six-channel small animal treadmill (Ugo Basile, Comerio, Italy). In the ILT, mice began running at an initial speed of 6 m/min, and the speed was increased by 3 m/min every 3 min until exhaustion. During the test, pulsed electrical stimulation (constant current mode, 0.3 mA intensity, 1 Hz frequency, 200 ms single pulse duration) was applied to the tail via the treadmill’s tail electrical stimulation grid to motivate the mice to continue running, and exhaustion was defined as receiving more than five electrical shocks in 1 min. EV was calculated using the formula: EV = *V* + (*n*/*b*) × *a*, where *V* is the speed of the last completed stage, n is the time spent in the incomplete stage, b is the stage duration, and a is the acceleration (21).

### 2.4 Overtraining model establishment

The downhill running-based overtraining protocol adopted in this study is a classic, widely validated model for mouse OTS research in exercise physiology, which stably induces the preclinical pathological state of OTS with high modeling success rate and reproducibility (22). The exercise intensity of the entire protocol was calibrated by exhaustion velocity (EV), with the calculation method following the recognized gold standard in the field, ensuring the standardization and reliability of the modeling process. Mice underwent 5 days of training and 2 days of rest each week. In the first 4 weeks (Phase 1), the exercise intensity was set at 60% of EV, with a duration of 60 min per day. During this phase, treadmill training was conducted at 0% incline. In the fifth week, the exercise intensity and duration remained unchanged, but the treadmill incline was adjusted to −14%, which was maintained until the end of the experiment. In the sixth week, the exercise intensity was increased to 70% of EV, while the duration remained 60 min per day. In the seventh week, the intensity was further increased to 75% of EV, and the duration was extended to 75 min. During the eighth week, the intensity and duration remained the same as in the seventh week, but mice completed two training sessions per day, with a 4-h interval between sessions. The detailed exercise protocol is shown in Table 1.

**Table 1:** Mice treadmill running protocol.

Experimental stage	Week	Incline gradient (%)	Velocity (% EV)	Time (min)
First stage	1	0	60	60
	2	0	60	60
	3	0	60	60
	4	0	60	60
Second stage	5	−14	60	60
	6	−14	70	60
	7	−14	75	75
	8	−14	75	75

### 2.5 Metabolic monitoring of experimental animals

Outside of exercise periods, the mice were housed in the SABLE Promethion metabolic and behavioral monitoring system for small animals, which uses a pull-mode gas measurement method to monitor real-time metabolic parameters, including oxygen consumption, carbon dioxide production, water intake, food intake, and spontaneous activity. Each monitoring period lasted 24 h, during which we analyzed the mice’s recovery phase following exercise.

### 2.6 Liver histopathological examination

Mouse liver tissues were fixed in 10% neutral formalin, followed by paraffin embedding and hematoxylin–eosin (HE) staining. Stained liver sections were imaged using an automated digital slide scanning system (3D Histech, Hungary).

### 2.7 Serum transaminase measurement

Blood samples were collected via orbital sinus puncture and left at room temperature (22 ± 2 °C) for 1 h. Afterward, the samples were centrifuged at 4 °C for 10 min to obtain serum supernatant. Serum alanine aminotransferase (ALT) and aspartate aminotransferase (AST) levels were measured using enzyme-linked immunosorbent assay (ELISA) according to the kit instructions.

### 2.8 Hepatic ROS detection

ROS levels in mouse liver tissue were measured using the DHE probe. Frozen liver sections were rinsed with washing solution at room temperature, followed by incubation with DHE probe solution in the dark. After nuclear staining with DAPI, slides were sealed and imaged using a digital slide scanner (Hamamatsu, Japan).

### 2.9 Measurement of oxidative stress markers in liver

Approximately 100 mg of liver tissue was excised and minced with surgical scissors. The tissue was homogenized with 900 μL of normal saline. The homogenates were centrifuged at 4 °C for 10 min, and the supernatant was collected. Liver oxidative stress markers, including malondialdehyde (MDA), superoxide dismutase (SOD) and glutathione (GSH), were measured following the instructions provided in the assay kits.

### 2.10 Ultrastructural analysis of liver mitochondria

Fresh liver tissue (1 mm^3^) was quickly fixed in 2.5% glutaraldehyde solution (in phosphate buffer, pH 7.4), followed by fixation in 1% osmium tetroxide. The samples were then dehydrated using a graded ethanol series and embedded in Epon 812 resin. Ultrathin sections were prepared using an ultramicrotome (Leica UCT, Germany). The sections were stained with uranyl acetate and lead citrate, and observed using a transmission electron microscope (HT7700, Japan).

### 2.11 Mitochondrial membrane potential assessment

Frozen sections were thawed at room temperature and circled with a PAP pen. Diluted JC-1 solution was added, and the sections were incubated at 37 °C for 20 min. Afterward, the sections were washed three times with PBS (5 min each time), sealed, and imaged using a digital slide scanner (Hamamatsu, Japan).

### 2.12 Western blot analysis

Liver tissue samples (20–30 mg) were homogenized in lysis buffer on ice and incubated for 30 min at 4 °C. For separating cytoplasmic and nuclear proteins, we followed the instructions provided in the kit (Beyotime, China). Protein concentration in the supernatant was determined using a BCA protein assay kit (Beyotime, China). Equal amounts of protein (60–80 μg) were loaded onto 8, 10, and 12% SDS-PAGE gels for electrophoresis. Proteins were transferred to polyvinylidene fluoride (PVDF) membranes using a transfer system (Bio-Rad, USA). After blocking with BSA, the membranes were incubated with primary and secondary antibodies. Imaging was performed using an ECL chemiluminescence detection system (Azure C300, USA).

### 2.13 Immunofluorescence co-localization analysis

Frozen sections were thawed at room temperature and circled with a PAP pen. The sections were permeabilized with Triton X-100 and blocked with BSA. They were then incubated with two different primary antibodies followed by the corresponding secondary antibodies. After nuclear staining with DAPI, the slides were sealed and imaged using a digital slide scanner (Hamamatsu, Japan).

### 2.14 Statistical data analysis

All experimental data are expressed as mean ± standard deviation (SD), where n represents independent biological replicates for all experiments, and technical replicates were only used for detection stability verification and not included in statistical analysis. For parameters such as exhaustive exercise time (EV) and body weight, repeated measures ANOVA was applied to analyze the overall inter-group differences and time-group interaction effects. For multi-group end-point indicators including Western blot and immunofluorescence staining, one-way ANOVA was performed using SPSS 19.0 (SPSS Inc., Chicago, IL, USA). *Post-hoc* tests were selected based on the comparison purpose: Tukey’s test was used for full pairwise comparisons between multiple groups when statistically significant differences were found between groups, and Dunnett’s test was employed specifically for comparisons between each experimental group and the control group. Semi-quantitative analysis of Western blot bands and immunofluorescence staining results was conducted using Image J software, and bar graphs were generated using GraphPad Prism 9.0 (GraphPad, La Jolla, CA, USA).

## 3 Results

### 3.1 Effects of Gs-Rb1 on exercise performance in OT-treated mice

**Figure 2:**
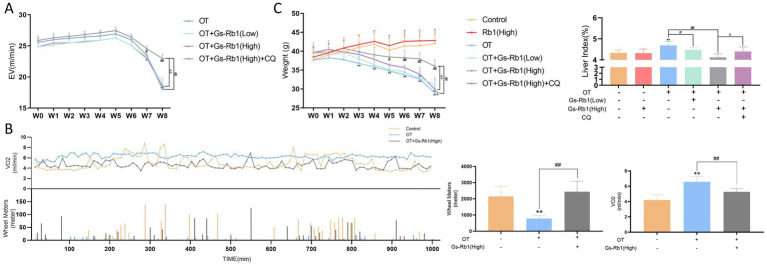
Effects of Gs-Rb1 on exercise performance, oxygen consumption, body weight, and liver index in OT-treated mice. **(A)** Longitudinal changes in EV values of ICR mice over eight consecutive weeks; **(B)** oxygen consumption and spontaneous activity (wheel running distance) during the recovery period on the last day of week 8; **(C)** changes in body weight of ICR mice over 8 weeks, and liver index calculated after sacrifice. Mean ± SD, *n* = 10 independent biological replicates per group. For the repeated observation indicators (EV and body weight), statistical analysis was performed by repeated measures ANOVA; for other end-point indicators, one-way ANOVA followed by Tukey’s *post-hoc* test was used for multi-group comparisons. *p < 0.05 vs. control, **p < 0.01 vs. control, #*p* < 0.05 vs. OT group, ##*p* < 0.01 vs. OT group, ^*p* < 0.05 vs. OT + Gs-Rb1 (High) group, ^^*p* < 0.01 vs. OT + Gs-Rb1 (High) group.

We conducted an ILT before the start of the experiment and after the end of each weekly training session to calculate EV, a widely used parameter in evaluating exercise performance in small animals. The results (Figure 2A) showed that mice in all groups exhibited adaptive enhancements in treadmill performance during the first 5 weeks, with EV values peaking at the end of week five. Repeated measures ANOVA revealed significant intra- and intergroup effects, indicating that starting from the end of week seven, the OT + Gs-Rb1 (High) group exhibited a significant difference compared with the OT group (*p* < 0.05), with the difference becoming more pronounced by the end of week eight (*p* < 0.01). The addition of CQ diminished this difference, and low-dose Gs-Rb1 did not produce significant effects.

### 3.2 Effects of Gs-Rb1 on resting oxygen consumption in OT-treated mice

Outside of training hours, we continuously monitored the gas metabolism of the mice. Since oxygen consumption is highly correlated with the physical activity of the mice, we also recorded their spontaneous running behavior post-exercise. As shown in Figure 2B, spontaneous activity (wheel running distance) in the OT group was significantly lower than that in the Control group, with almost no spontaneous activity (*p* < 0.01). After treatment with high-dose Gs-Rb1, mice in the OT group showed a significant increase in spontaneous activity (*p* < 0.01).

Next, we analyzed oxygen consumption during the recovery period after training, focusing on the Control, OT, and OT + Gs-Rb1 (High) groups. The analysis revealed that the oxygen consumption in the Control and OT + Gs-Rb1 (High) groups fluctuated in a wave-like pattern, which was directly related to spontaneous activity reflected by wheel running distance. However, in the OT group, oxygen consumption remained relatively stable with no significant fluctuations, consistent with the almost absent spontaneous activity in this group. When analyzing resting oxygen consumption across the groups, we found that the OT group had significantly higher oxygen consumption than both the Control and OT + Gs-Rb1 (High) groups (*p* < 0.01).

### 3.3 Effects of Gs-Rb1 on body weight and liver index in OT-treated mice

Prior to the experiment, we measured the body weight of mice in each group, and no statistically significant differences were observed. Subsequently, weekly body weight measurements were conducted, and repeated measures ANOVA was used to assess intergroup differences. As shown in Figure 2C, after 8 weeks of feeding, mice in both the Control group and the Gs-Rb1 alone-treated group exhibited a gradual increase in body weight. Significant differences were observed within each group (between pre-training and post-week 8 measurements) (*p* < 0.05), though no significant differences were found between the two groups (*p* > 0.05). In contrast, body weight in the remaining four groups demonstrated a downward trend, with the OT group and OT + Gs-Rb1 (High) + CQ group experiencing the most pronounced weight loss (*p* < 0.01). Beginning in week three, the body weight of trained mice began to diverge significantly from the Control group. As training intensity increased, body weight consistently declined. By weeks seven and eight, the OT group, OT + Gs-Rb1 (Low) group, and OT + Gs-Rb1 (High) + CQ group exhibited accelerated weight loss, while the OT + Gs-Rb1 (High) group displayed a significant difference compared to both the OT group and OT + Gs-Rb1 (High) + CQ group (*p* < 0.01).

The liver index is commonly used to assess liver health, where an elevated index may indicate liver tissue damage such as congestion, edema, fatty degeneration, or hypertrophy, while a reduced liver index may suggest liver atrophy or other degenerative changes. As shown in Figure 2C, the liver index of the OT group increased significantly compared to the Control group (*p* < 0.01). However, mice in the OT + Gs-Rb1 (High) group exhibited a significant reduction in liver index compared to the OT group (*p* < 0.01). This reduction was reversed in the OT + Gs-Rb1 (High) + CQ group, where the liver index increased once again (*p* < 0.01).

### 3.4 Effects of Gs-Rb1 on liver injury induced by OT in mice

**Figure 3:**
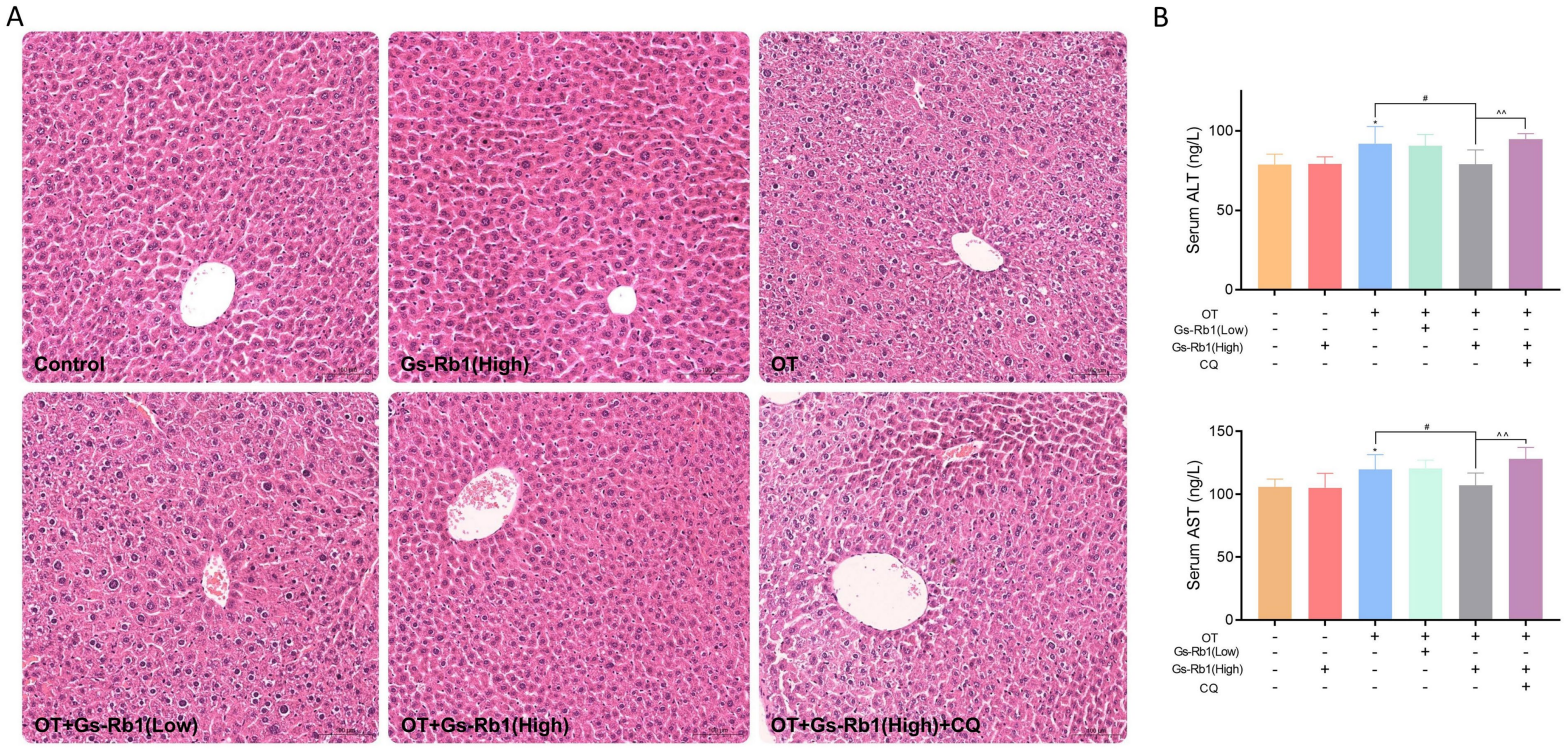
Effects of Gs-Rb1 on liver damage in OT-treated mice. **(A)** Representative histopathological images of liver tissues via HE staining (magnification ×200); **(B)** levels of liver injury markers ALT and AST in serum. All data are presented as mean ± SD, *n* = 10 independent biological replicates per group. Statistical analysis was performed by one-way ANOVA followed by Tukey’s *post-hoc* test. *p < 0.05 vs. control group, #*p* < 0.05 vs. OT group, ^*^p* < 0.01 vs. OT + Gs-Rb1 (High) group.

Liver histopathology is an important method for visually assessing liver injury. Through HE staining, we observed that the liver tissue structures in the Control group and the Gs-Rb1 alone-treated group were similar, with nuclei of uniform size and distribution, clear hepatocyte contours, and hepatic cords radiating from the central vein. In the OT group, significant hepatocyte edema, disorganized hepatic cords, and focal hepatocyte necrosis were evident. Compared to the OT group, the liver tissue of mice in the OT + Gs-Rb1 (low) group showed some alleviation of edema, though the hepatic cord network remained unclear, and focal hepatocyte necrosis was still prominent. In contrast, the OT + Gs-Rb1 (High) group exhibited a marked reduction in liver damage, with clear hepatic cord structure, and no significant hepatocyte edema or necrosis. The protective effect of Gs-Rb1 disappeared when CQ was added, with cellular damage reappearing (Figure 3A).

We then verified the histopathological results by measuring the concentrations of liver injury markers (serum ALT and AST). As shown in Figure 3B, Gs-Rb1 alone had no significant effect on serum ALT and AST levels in ICR mice. OT significantly increased the levels of ALT and AST in the serum (*p* < 0.05). High-dose Gs-Rb1 effectively attenuated the overtraining-induced increase in serum ALT and AST (*p* < 0.05), while low-dose Gs-Rb1 showed no significant effect. Additionally, we found that the protective effect of Gs-Rb1 disappeared with the addition of CQ, resulting in a significant elevation in serum ALT and AST levels once again (*p* < 0.01).

### 3.5 Effects of Gs-Rb1 on hepatic ROS and oxidative stress markers in OT-treated mice

To investigate the changes in oxidative stress status in the liver of OT-treated mice, we measured the levels of ROS and other oxidative stress-related markers in liver tissue.

**Figure 4:**
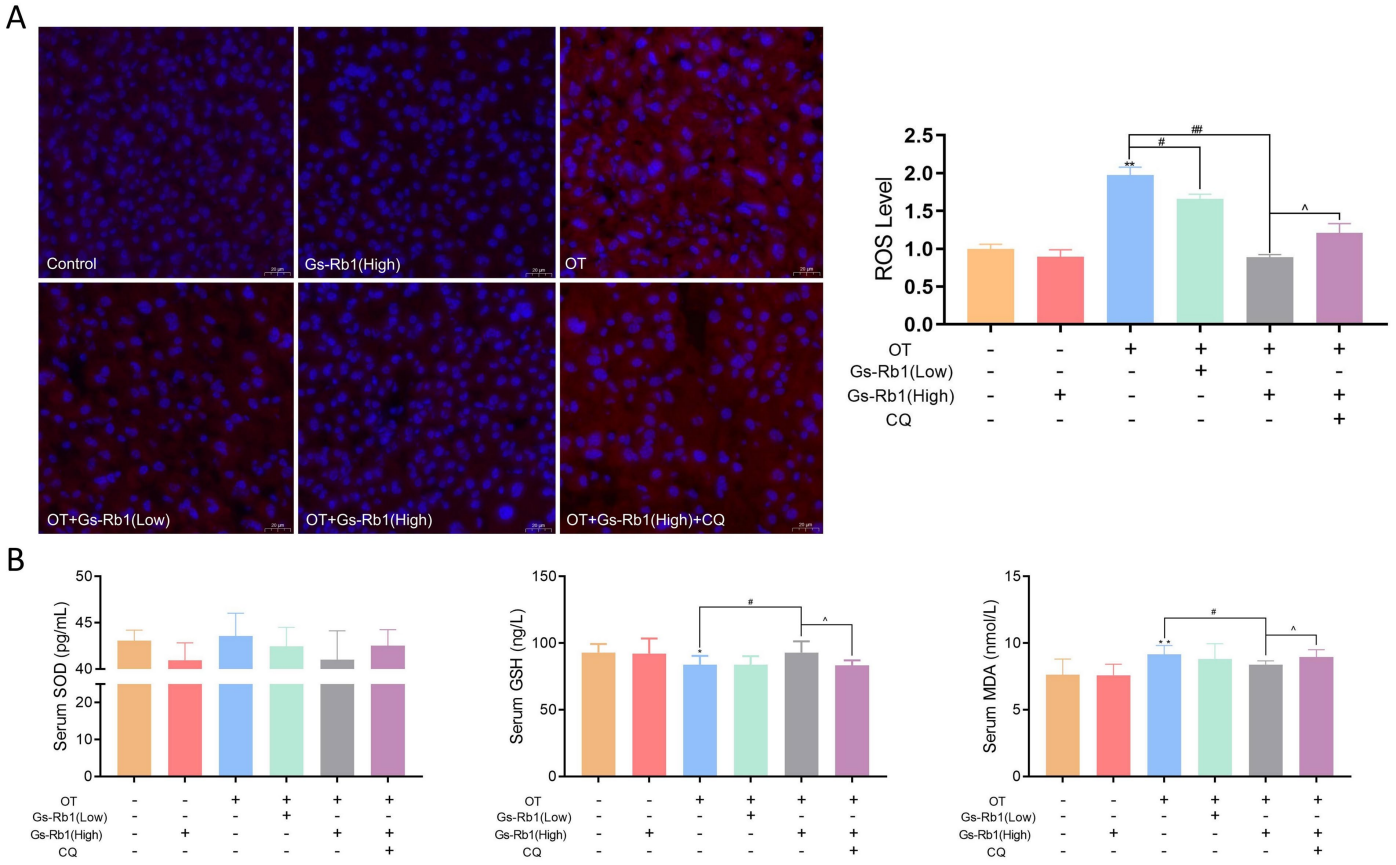
Effects of Gs-Rb1 on hepatic ROS levels and oxidative stress-related markers in OT-treated mice. **(A)** Representative fluorescent images of frozen liver sections to assess ROS levels via DHE staining; **(B)** levels of MDA, SOD, and GSH in liver tissue. All data are presented as mean ± SD, *n* = 10 independent biological replicates per group. Statistical analysis was performed by one-way ANOVA followed by Tukey’s *post-hoc* test. *p < 0.05 vs. control group, **p < 0.01 vs. control group, #*p* < 0.05 vs. OT group, ##*p* < 0.01 vs. OT group, ^*p* < 0.05 vs. OT + Gs-Rb1 (High) group.

First, we used Dihydroethidium (DHE) probe on frozen liver sections of the mice. The DHE probe is oxidized by ROS in the tissue, producing a red fluorescent signal, with the intensity of the fluorescence directly proportional to the ROS levels. Observation of the stained images (Figure 4A) revealed significant accumulation of red fluorescence in the liver tissue of OT-treated ICR mice (*p* < 0.01), indicating elevated ROS levels. Under the protective effect of Gs-Rb1, the red fluorescence was markedly reduced, with the high-dose Gs-Rb1 group showing a stronger effect (*p* < 0.01). The addition of CQ reversed this effect, with ROS-related red fluorescence signal significantly increased again (*p* < 0.05).

We further measured the levels of MDA, total SOD, and reduced GSH in the liver tissue of ICR mice to comprehensively evaluate hepatic oxidative stress status. As shown in Figure 4B, Gs-Rb1 treatment alone had no significant effect on these three oxidative stress markers compared with the control group. However, OT exposure significantly increased hepatic MDA content (*p* < 0.01) and decreased hepatic GSH level (*p* < 0.05), indicating that chronic overtraining induced distinct oxidative stress damage in the mouse liver. Compared with the OT group, the OT + Gs-Rb1 (High) group showed significantly reduced MDA content (*p* < 0.05) and elevated GSH level (*p* < 0.05), suggesting that high-dose Gs-Rb1 effectively alleviated OT-induced hepatic oxidative stress damage. The low-dose Gs-Rb1 group presented a similar ameliorative trend, but the changes did not reach statistical significance. No statistically significant difference in hepatic total SOD activity was observed among all experimental groups, despite a mild upward trend in the OT + high-dose Gs-Rb1 group. Additionally, CQ treatment significantly reversed the ameliorative effect of Gs-Rb1 on the above oxidative stress markers (*p* < 0.05).

### 3.6 Effects of Gs-Rb1 on mitochondrial damage in the liver of OT-treated mice

**Figure 5:**
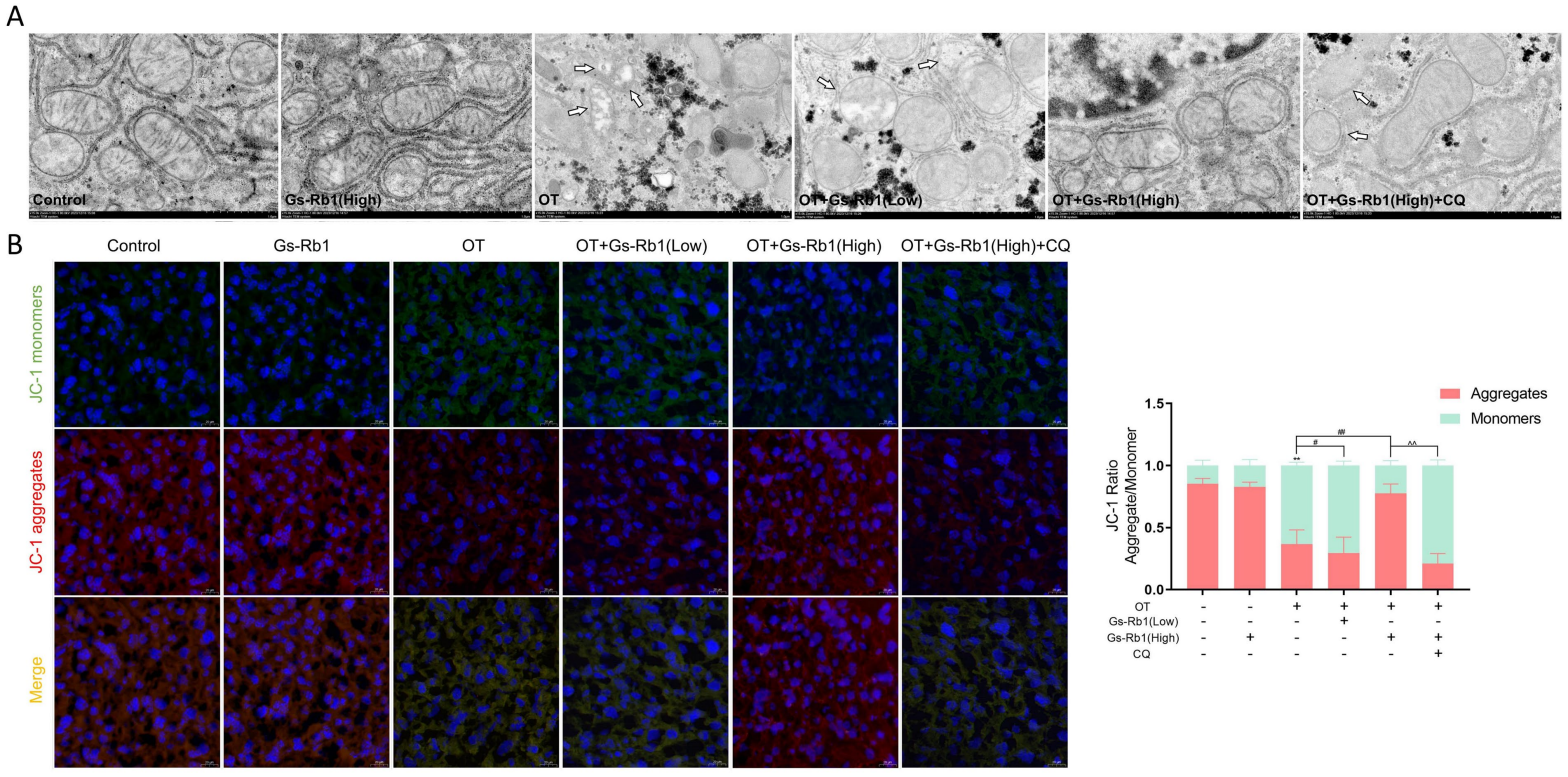
Effects of Gs-Rb1 on mitochondrial damage in the liver of OT-treated mice. **(A)** Representative transmission electron microscopy images showing mitochondrial morphology in liver tissues (magnification × 1,500), with damaged mitochondria marked by white arrows; **(B)** representative fluorescent images and quantitative analysis of mitochondrial membrane potential detected using the JC-1 fluorescent probe. All data are presented as mean ± SD, *n* = 3 independent biological replicates per group. Statistical analysis was performed by one-way ANOVA followed by Tukey’s *post-hoc* test. **p < 0.01 vs. control group, #*p* < 0.05 vs. OT group, ##*p* < 0.01 vs. OT group, ^^*p* < 0.01 vs. OT + Gs-Rb1 (High) group.

To verify whether excessive ROS is accompanied by mitochondrial damage, we observed and assessed the condition of the mitochondria. First, we examined the ultrastructure of hepatocytes using transmission electron microscopy and found that the mitochondria in the liver cells of OT-treated mice exhibited significant swelling, vacuolization, disrupted cristae, and even ruptured mitochondrial membranes. Gs-Rb1 alleviated this mitochondrial damage caused by OT, with the high-dose Gs-Rb1 group showing more pronounced effects (Figure 5A).

Next, we assessed mitochondrial membrane potential using the JC-1 fluorescent probe. In healthy mitochondria, JC-1 forms aggregates that emit red fluorescence, whereas with decreased membrane potential, JC-1 exists as monomers emitting green fluorescence; mitochondrial health was evaluated by analyzing the red-to-green fluorescence ratio. Frozen section staining results showed that in the Control group and the Gs-Rb1 alone-treated group, green fluorescence was minimal and red fluorescence was strong. In the OT group, a significant increase in green fluorescence and a decrease in red fluorescence were observed (*p* < 0.01). This change was alleviated by Gs-Rb1 treatment, with a more significant protective effect detected in the high-dose group (*p* < 0.01). However, with the addition of CQ, green fluorescence increased again and red fluorescence decreased significantly (*p* < 0.01) (Figure 5B).

### 3.7 Effects of Gs-Rb1 on mitophagy in OT-treated mice

To explore whether Gs-Rb1 alleviates mitochondrial damage via regulating autophagy, we detected autophagic flux and mitophagy-related markers in mouse liver tissues.

**Figure 6:**
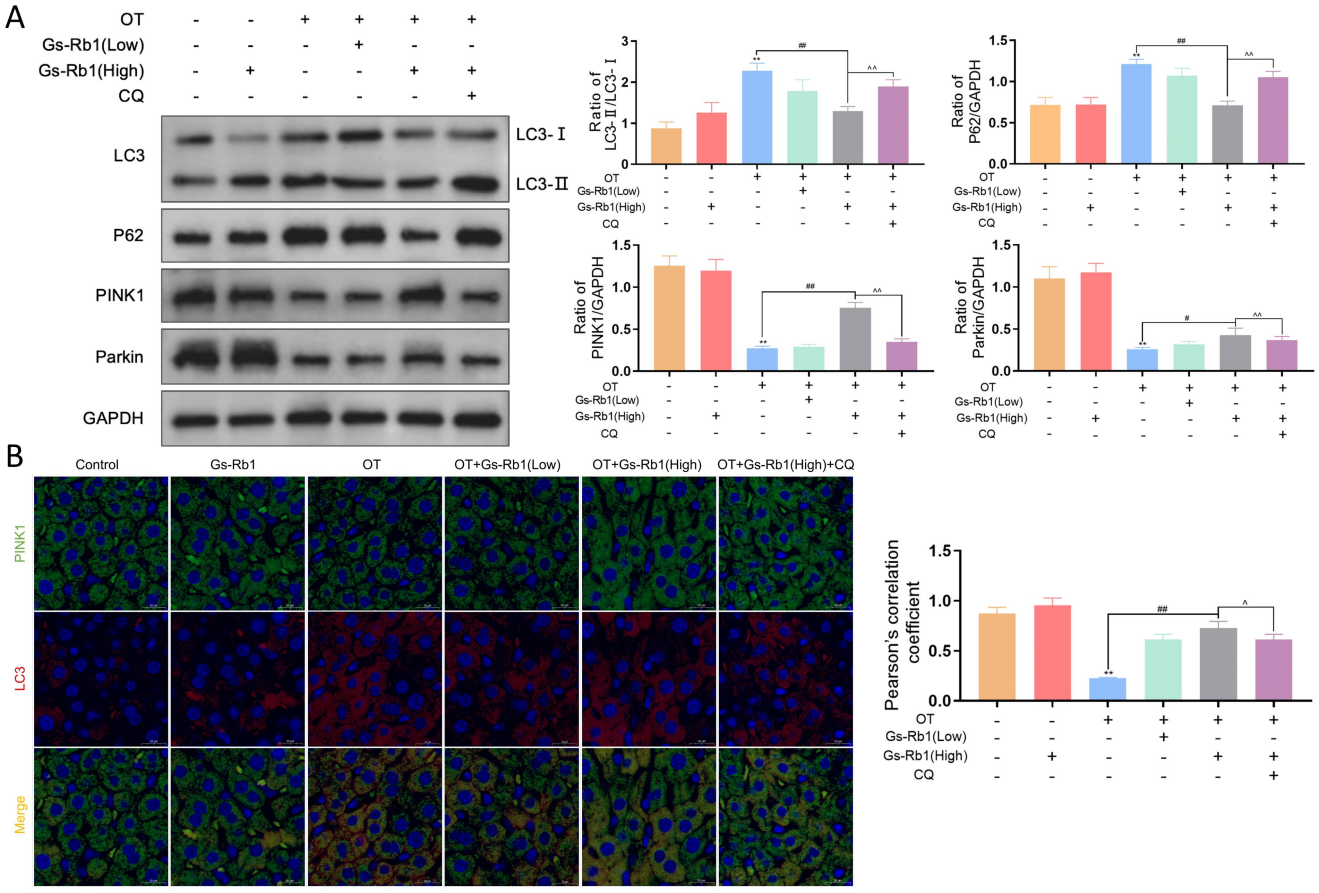
Effects of Gs-Rb1 on mitophagy in OT-treated mice. **(A)** Representative western blot images showing the expression of mitophagy-related proteins LC3, P62, PINK1, and Parkin; **(B)** representative double immunofluorescence images of LC3-PINK1 colocalization to assess the fusion of autophagosomes and mitochondria, with Pearson’s correlation coefficient analyzed for at least 50 cells per sample. All data are presented as mean ± SD, *n* = 3 independent biological replicates per group. Statistical analysis was performed by one-way ANOVA followed by Tukey’s *post-hoc* test. *p* < 0.01 vs. control group, ^#^*p* < 0.05 vs. OT group, ^##^*p* < 0.01 vs. OT group, ^*p* < 0.05 vs. OT + Gs-Rb1 (High) group, ^^*p* < 0.01 vs. OT + Gs-Rb1 (High) group.

First, we evaluated autophagic flux by Western blot analysis of LC3 (autophagosome marker) and P62 (autophagic degradation substrate), with the results shown in Figure 6A. Compared with the control group, the LC3-II/LC3-I ratio was significantly increased in all experimental groups (*p* < 0.01). Compared with the control group, OT treatment induced a simultaneous significant elevation of both the LC3-II/LC3-I ratio and P62 protein levels (*p* < 0.01). Compared with the OT group, the OT + Gs-Rb1 (High) group showed a significant reduction in both the LC3-II/LC3-I ratio and P62 levels (*p* < 0.01). Compared with the OT + Gs-Rb1 (High) group, the OT + Gs-Rb1 (High) + CQ group exhibited a significant increase in the LC3-II/LC3-I ratio (*p* < 0.05) and P62 protein levels (*p* < 0.01). Compared with the OT group, no significant differences in the LC3-II/LC3-I ratio or P62 expression were observed in the OT + Gs-Rb1 (Low) group, and Gs-Rb1 treatment alone showed no significant effect on P62 expression or basal autophagic flux in normal mice.

We further examined the expression of PINK1 and Parkin, the core proteins related to mitophagy initiation, via Western blot. Our results showed that, compared with the control group, OT treatment significantly downregulated PINK1 and Parkin expression in mouse liver (*p* < 0.01). Compared with the OT group, Gs-Rb1 upregulated both proteins in a dose-dependent manner, with the most prominent effect observed in the high-dose Gs-Rb1 group (*p* < 0.01), and a significant effect also detected in the low-dose Gs-Rb1 group (*p* < 0.05). Compared with the OT + Gs-Rb1 (High) group, CQ treatment significantly abolished the upregulation of PINK1 and Parkin induced by Gs-Rb1 (*p* < 0.01).

To directly observe the recruitment of autophagosomes to impaired mitochondria, we performed immunofluorescence co-localization assay of PINK1 and LC3. PINK1 (labeled with green fluorescence) and LC3 (labeled with red fluorescence) co-localized puncta were used to reflect the key event in mitophagy initiation. As shown in Figure 6B, compared with the control group, the OT group exhibited a significant decrease in the number of PINK1-LC3 co-localized puncta (*p* < 0.01). Compared with the OT group, Gs-Rb1 treatment increased the co-localization of PINK1 and LC3 in a dose-dependent manner, with the most significant effect detected in the OT + Gs-Rb1 (High) group (*p* < 0.01). Compared with the OT + Gs-Rb1 (High) group, CQ co-treatment significantly reversed the Gs-Rb1-induced increase in PINK1-LC3 co-localization (*p* < 0.05).

### 3.8 Effects of Gs-Rb1 on Nrf2 expression and nuclear translocation in OT-treated mice

Nrf2 is the core transcription factor governing cellular redox homeostasis and mitochondrial quality control. We isolated cytoplasmic and nuclear proteins from mouse liver tissues to detect Nrf2 nuclear translocation via Western blot, and further used ML385, a specific Nrf2 inhibitor, to validate the role of Nrf2 in the hepatoprotective effects of Gs-Rb1.

**Figure 7:**
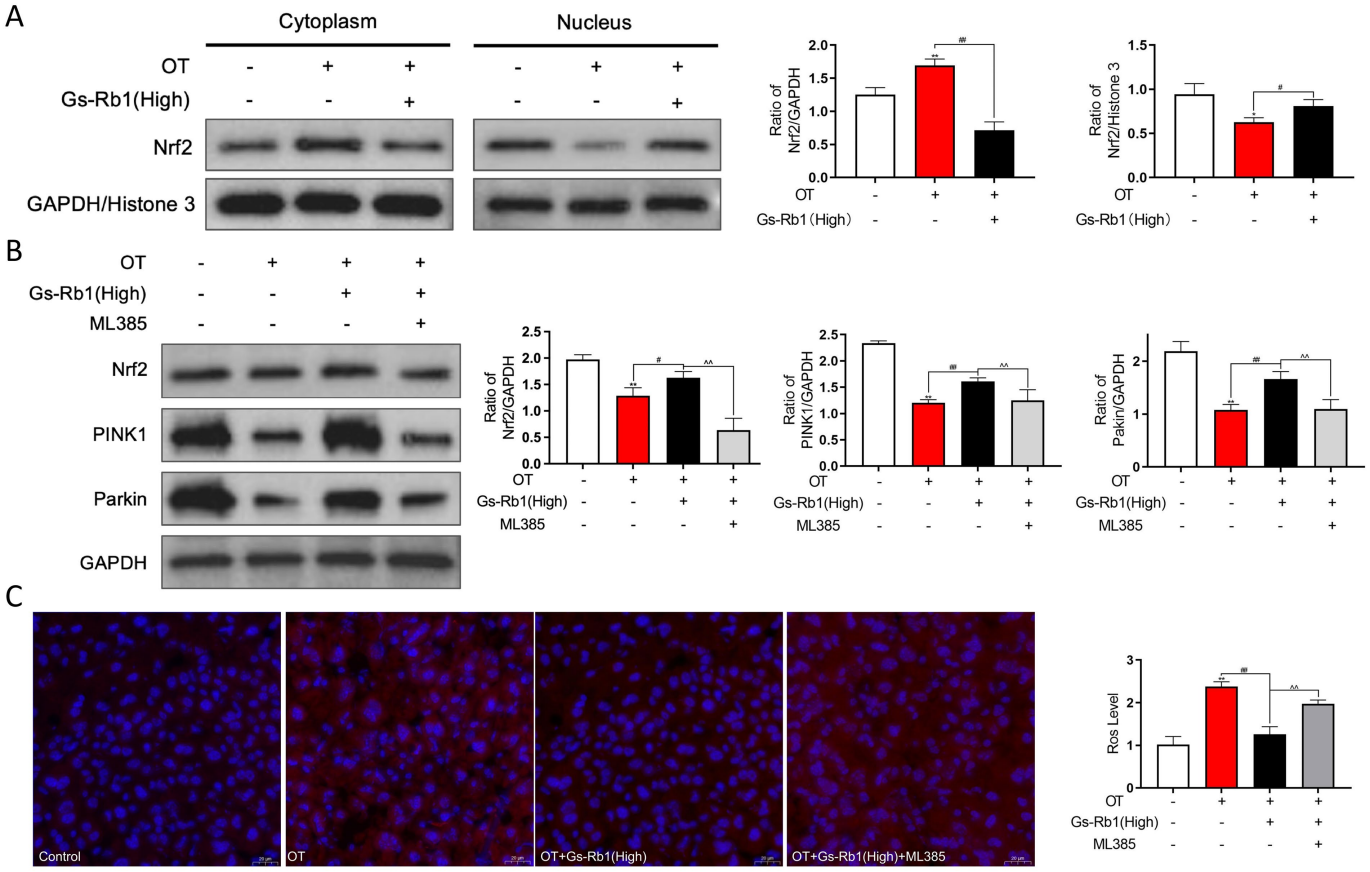
Effects of Gs-Rb1 on Nrf2 expression and nuclear translocation in OT-treated mice. **(A)** Representative western blot images showing the expression of Nrf2 protein in nuclear and cytoplasmic fractions of liver tissues; **(B)** representative western blot images showing the effects of Gs-Rb1 and ML385 on the expression of Nrf2, PINK1, and Parkin in the liver of OT-treated mice; **(C)** representative fluorescent images and quantitative analysis of ROS levels in mouse liver tissues detected by DHE fluorescent probe. All data are presented as mean ± SD, *n* = 3 independent biological replicates per group. Statistical analysis was performed by one-way ANOVA followed by Tukey’s *post-hoc* test. **p < 0.01 vs. control, ##*p* < 0.01 vs. OT group, ^^*p* < 0.01 vs. OT + Gs-Rb1 (High) group.

As shown in Figure 7A, OT treatment significantly reduced nuclear Nrf2 expression in mouse liver (*p* < 0.05), and this inhibitory effect was alleviated by Gs-Rb1 treatment (*p* < 0.05). The expression trend of Nrf2 in the cytoplasm was opposite to that in the nucleus.

We then analyzed the expression of Nrf2 and mitophagy-related proteins PINK1 and Parkin after ML385 intervention via Western blot (Figure 7B). The results showed that, compared with the control group, OT treatment significantly downregulated Nrf2 expression in mouse hepatocytes (*p* < 0.01), while Gs-Rb1 treatment significantly restored Nrf2 expression (*p* < 0.01). ML385 treatment significantly suppressed the Gs-Rb1-induced upregulation of Nrf2 (*p* < 0.01), and also inhibited the expression of PINK1 and Parkin (*p* < 0.01).

We further analyzed the effect of ML385 on hepatic ROS levels. As shown in Figure 7C, ML385 treatment significantly reversed the inhibitory effect of Gs-Rb1 on hepatic ROS accumulation, with ROS levels significantly increased again (*p* < 0.01).

## 4 Discussion

OT-induced liver health impairment is a high-incidence adverse health outcome in competitive sports and mass fitness, and the core nutritional and physiological basis of this impairment is the vicious cycle of mitochondrial damage and ROS overproduction, which further triggers oxidative stress and inflammatory cascade to aggravate hepatic injury (23). However, there is currently no recognized safe and effective nutritional strategy for its prevention and management. Recently, food-derived bioactive compounds from medicinal and edible homologous plants have received widespread attention for their excellent antioxidant, anti-inflammatory properties and high safety in alleviating chronic stress-induced organ injury (24). In this study, we systematically investigated the protective effect of food-derived Gs-Rb1, a bioactive component from medicinal and edible homologous ginseng, against chronic OT-induced liver health impairment in mice. Our results revealed that high-dose Gs-Rb1 (40 mg/kg b.w.) significantly mitigated OT-induced systemic fatigue and hepatic pathological damage, and this hepatoprotective effect was fully dependent on the activation of Nrf2-mediated PINK1/Parkin mitophagy. Specifically, Gs-Rb1 promoted Nrf2 nuclear translocation, activated PINK1/Parkin-mediated selective mitophagy, restored impaired autophagic flux, and maintained mitochondrial homeostasis. This conclusion was solidly supported by the reversal of Gs-Rb1’s protective effects via the autophagy inhibitor CQ and Nrf2-specific inhibitor ML385.

In terms of overall physical performance, chronic overtraining markedly impaired exercise capacity, accelerated fatigue accumulation, induced body weight loss, and elevated resting oxygen uptake in mice, which were effectively alleviated by high-dose Gs-Rb1 intervention. OTS is characterized by persistent sympathetic overactivation and systemic metabolic disturbance after long-term excessive training with insufficient recovery (25). As the central metabolic hub during exercise, hepatic homeostasis directly sustains whole-body energy supply via glycogen metabolism and systemic metabolic regulation. Persistent hepatic injury under overtraining conditions disrupts energy metabolism homeostasis, while sympathetic hyperactivation-mediated elevation of resting metabolic rate further increases hepatic metabolic burden, forming a vicious cycle that exacerbates exercise intolerance and chronic fatigue. Accordingly, the hepatoprotective effects of Gs-Rb1 directly support systemic metabolic stability, contributing to the improvement of exercise performance and resistance to OT-induced fatigue.

OT induced a significant elevation in liver index, accompanied by hepatocyte edema, disorganized hepatic cords, focal necrosis, and increased serum levels of ALT and AST, which are classic pathological manifestations of exercise-induced liver injury. The elevated liver index under OT was mainly attributed to hepatocyte edema and inflammatory infiltration secondary to oxidative stress. Consistently, our hepatic oxidative stress assays confirmed that OT significantly increased the lipid peroxidation product MDA and depleted the endogenous antioxidant GSH, with high-dose Gs-Rb1 markedly reversing these changes in a dose-dependent manner; no significant difference in SOD activity was detected across groups, which is consistent with previous studies showing that long-term chronic overtraining may exhaust the compensatory upregulation of endogenous antioxidant enzymes in the liver (26). These results confirmed that Gs-Rb1 effectively alleviated OT-induced hepatic histomorphological and biochemical abnormalities, laying a solid phenotypic foundation for its application as a dietary nutritional intervention.

Nrf2 is the master transcription factor governing cellular redox homeostasis, and is a well-established target for natural antioxidants to mitigate chronic stress-induced oxidative damage (27–29). Under physiological conditions, Nrf2 is sequestered in the cytoplasm by Keap1 for proteasomal degradation; under oxidative stress, it translocates to the nucleus to activate the endogenous antioxidant defense system via antioxidant response element (ARE) binding. While existing exercise-related studies have mainly focused on this canonical “passive ROS clearance” function of Nrf2, its role in regulating mitochondrial quality control via mitophagy in OT-induced liver injury remains poorly understood. Nuclear translocation is the prerequisite and gold-standard marker of Nrf2 pathway activation. Our nucleocytoplasmic protein separation assay provided direct evidence that Gs-Rb1 significantly promoted Nrf2 nuclear translocation in the liver of OT mice, characterized by a marked decrease in cytoplasmic Nrf2 protein level and a corresponding increase in nuclear Nrf2 level, confirming that Gs-Rb1 effectively activates the hepatic Nrf2 signaling pathway under chronic OT conditions. The PINK1/Parkin axis is the well-recognized core pathway governing selective clearance of damaged mitochondria, which initiates the mitophagy program by sensing mitochondrial oxidative damage, recruiting autophagic machinery to impaired mitochondria, and mediating their targeted lysosomal degradation (30, 31). Beyond direct antioxidant effects, Nrf2 can transcriptionally activate PINK1, the core initiator of mitophagy, to regulate mitochondrial homeostasis (32). Consistent with this, our results showed that Gs-Rb1 significantly upregulated the protein expression of PINK1 and Parkin in the liver of OT mice. Further validation with the Nrf2-specific inhibitor ML385 revealed that ML385 significantly inhibited the Gs-Rb1-induced upregulation of total Nrf2 protein, and simultaneously reversed the upregulation of PINK1 and Parkin mediated by Gs-Rb1. These observations suggest that Nrf2 may play an important role in mediating the regulatory effect of Gs-Rb1 on the PINK1/Parkin mitophagy axis. To our knowledge, this study is the first to demonstrate that Gs-Rb1 targets the Nrf2-PINK1/Parkin axis to alleviate chronic OT-induced liver injury, expanding the pathophysiological scope of Nrf2-mediated antioxidant defense in exercise stress. Our autophagic flux data further clarified the specific regulatory effect of Gs-Rb1 on mitophagy: OT induced simultaneous elevation of LC3-II/LC3-I ratio and P62 protein, indicating impaired autophagic degradation and autophagosome accumulation in the liver, while Gs-Rb1 significantly reduced both markers and restored autophagic flux. The PINK1-LC3 immunofluorescence co-localization assay provided direct cellular-level evidence that Gs-Rb1 promotes the recruitment of autophagosomes to damaged mitochondria, confirming that the pro-autophagic effect of Gs-Rb1 is targeted to impaired mitochondria, rather than non-specific bulk autophagy. Notably, the autophagy inhibitor CQ completely reversed the restorative effect of Gs-Rb1 on hepatic autophagic flux and its hepatoprotective effect, further verifying that mitophagy activation is indispensable for the beneficial effect of Gs-Rb1. These results collectively confirmed that Gs-Rb1 breaks the vicious cycle of “mitochondrial damage-ROS overproduction” under OT conditions via activating Nrf2-mediated mitophagy and restoring autophagic flux, which provides a novel “source control” antioxidant nutritional strategy distinct from the canonical passive ROS clearance pathway.

This mechanistic discovery also endows Gs-Rb1 with clear application advantages over mainstream sports nutritional supplements. Conventional exogenous antioxidants widely used in sports nutrition predominantly exert non-specific free radical-scavenging effects, failing to target the mitochondrial source of oxidative stress (33, 34); performance-focused supplements such as creatine and branched-chain amino acids mainly improve skeletal muscle function, with insufficient attention to the liver, the core metabolic hub of exercise. Unlike these agents, Gs-Rb1 exerts dual benefits: it enhances endogenous antioxidant capacity via Nrf2 activation, and simultaneously eliminates the root of oxidative stress via targeted regulation of mitophagy, while protecting hepatic homeostasis and improving exercise capacity, highlighting its unique application value in exercise health promotion.

This study has several limitations. First, we used the pharmacological inhibitor ML385 to verify the role of Nrf2, and while the dose and protocol we used are well-established in the field, we cannot fully exclude potential off-target effects. Thus, the absolute causal relationship between Nrf2 and the PINK1/Parkin pathway needs to be further verified in future studies using liver-specific Nrf2 knockout/overexpression models. Second, this study was conducted in mice, and the translatability of the results to human athletes requires validation in subsequent clinical trials.

In summary, this study confirmed that Gs-Rb1 exerts a significant protective effect against OT-induced hepatic oxidative stress via activating the Nrf2-PINK1/Parkin mitophagy axis and restoring autophagic flux. Our findings not only fill a key mechanistic gap in the pathogenesis of OT-induced liver injury and deepen the understanding of Nrf2-mediated antioxidant defense in exercise stress, but also provide a novel theoretical basis and high-safety dietary nutritional supplement candidate for the application of food-derived bioactive compounds in exercise-related health promotion.

## 5 Conclusion

**Figure 8:**
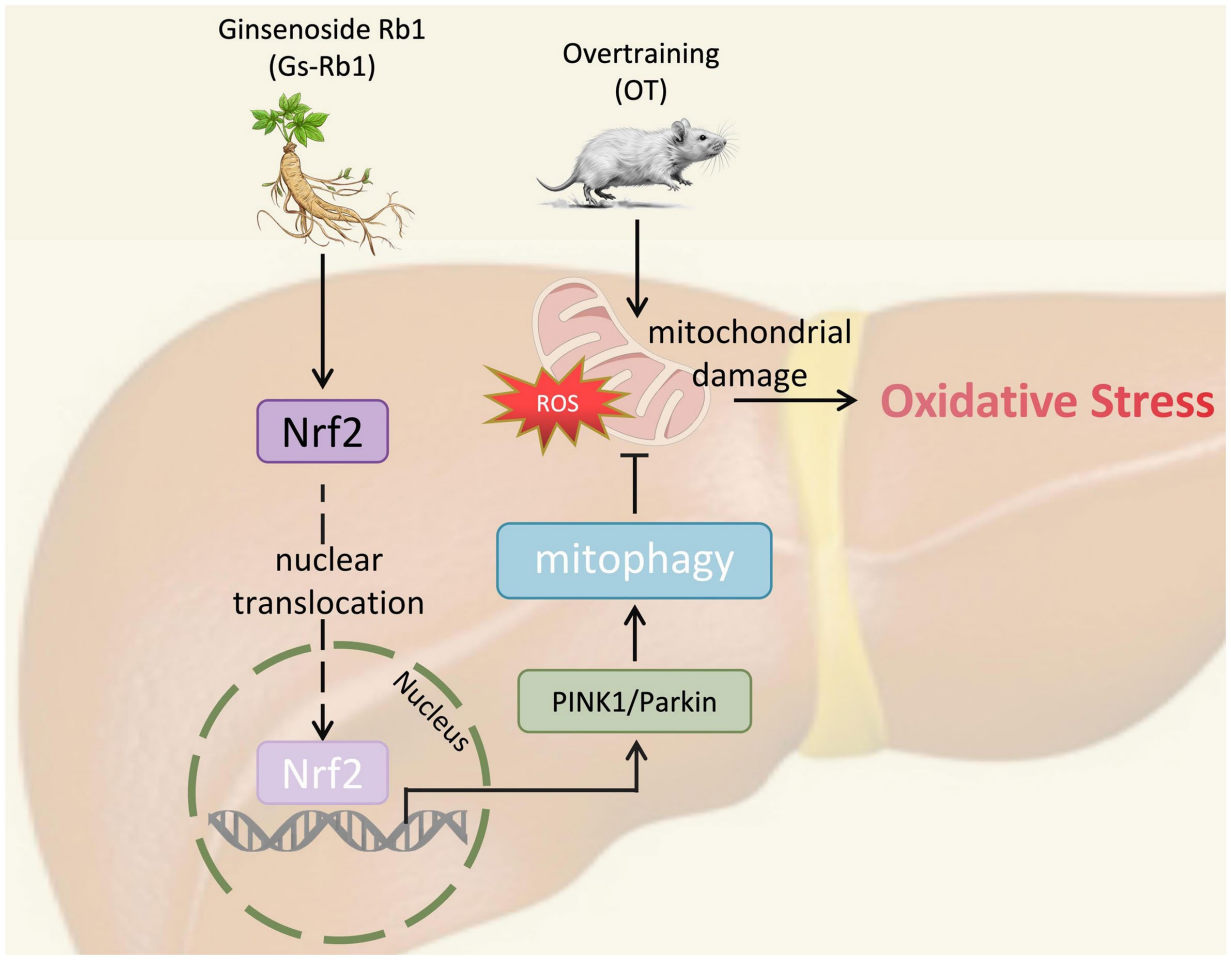
The mechanisms confirmed by this study.

In this study, we investigated the protective effects and underlying mechanisms of Gs-Rb1 against OT-induced oxidative stress and liver injury in mice, and identified Nrf2 as a key regulatory target. Our findings demonstrate that Gs-Rb1 enhances Nrf2 expression and promotes its nuclear translocation, which in turn activates PINK1/Parkin pathway-mediated mitophagy and restores impaired autophagic flux (Figure 8). This process facilitates the targeted clearance of damaged mitochondria, alleviates OT-induced hepatic oxidative stress and injury, and further improves exercise capacity and fatigue resistance in OT mice.

## Data Availability

The original contributions presented in the study are included in the article/supplementary material, further inquiries can be directed to the corresponding author.
